# Time Perspective and Emotion Regulation as Predictors of Age-Related Subjective Passage of Time

**DOI:** 10.3390/ijerph121215034

**Published:** 2015-12-17

**Authors:** Marc Wittmann, Tina Rudolph, Damisela Linares Gutierrez, Isabell Winkler

**Affiliations:** 1 Institute for Frontier Areas of Psychology and Mental Health, Wilhelmstr. 3a, Freiburg 79098, Germany; linares@igpp.de; 2 Department of Psychology, Forschungsmethodik und Evaluation, Chemnitz University of Technology, Chemnitz 09107, Germany; tina.rudolph@gmx.net (T.R.); isabell.winkler@phil.tu-chemnitz.de (I.W.)

**Keywords:** time perspective, ZTPI, FMI, time awareness, passage of time, aging, emotion regulation, mindfulness, balanced time perspective

## Abstract

Hardly any empirical work exists concerning the relationship between the intra-individually stable time perspective relating to the past, present, and future and the subjective speed of time passing in everyday life. Moreover, studies consistently show that the subjective passage of time over the period of the last ten years speeds up as we get older. Modulating variables influencing this phenomenon are still unknown. To investigate these two unresolved issues, we conducted an online survey with *n* = 423 participants ranging in age between 17 and 81 assessing trait time perspective of the past, present, and future, and relating these subscales with a battery of measures pertaining to the subjective passage of time. Moreover, the subjective passage of time as an age-dependent variable was probed in relationship to emotion awareness, appraisal and regulation. Results show how present hedonism is linked with having fewer routines in life and a faster passage of the last week; the past negative perspective is related to time pressure, time expansion and more routine; a pronounced future perspective is related to a general faster passage of time. Importantly, increased emotion regulation and a balanced time perspective are related to a slower passage of the last ten years. These novel findings are discussed within models of time perception and the time perspective.

## 1. Introduction

In a classification of time experiences at least three aspects can be discerned: (1) the *time perspective* of past, present, and future; (2) *time estimation* as measured by the accuracy in estimating clock time; and (3) *time awareness* as the subjective impression of time passing relatively fast or slow. Conceptually, it has repeatedly been argued that these three dimensions are interrelated.

To highlight one line of research, it has been shown that a more pronounced present perspective at the expense of the future perspective is associated with an inability to delay gratification, *i.e.*, individuals select choices with smaller but sooner rewards over those with larger but later rewards [1,2]. For most people the subjective value of future rewards decreases considerably with longer waiting time. Impulsive individuals have an even stronger “temporal myopia”, *i.e.*, only what is located within a shortened temporal horizon of the present moment is relevant [3]. That is, impulsive individuals who are present oriented are not as able to wait through a period of time as they act more on the spur of the moment—behaviour which in the long run can have negative consequences [4].

In order to explain this behaviour it has been suggested that a stronger focus on the momentary passage of time in more present-oriented, impulsive individuals would lead to a relative overestimation of duration (time estimation) and the feeling that time is passing more slowly (time awareness). The feeling of having to wait too long would consequently lead to short-sighted decision-making [5,6]. Individuals with psychiatric syndromes who are highly impulsive indeed overestimate duration in the seconds-to-minutes range [7,8]. In a different line of research experimentally induced social exclusion led to a less pronounced future perspective in individuals and a relative overestimation of temporal intervals of 40 and 80 s duration [9].

Ideally we flexibly switch the time perspective, either focusing on the past from the standpoint of the psychological present or imaging the future from the present perspective to create alternative goal states [10]. An impulsive state would mean that one would be stuck in the present moment at the expense of the future perspective [4]. Standing in contrast to the impulsive present perspective, another form of present-perspective is achieved through meditation techniques where individuals learn to focus on the present moment without being distracted by upcoming external stimuli and thoughts. Importantly, one has to differentiate between an impulsive present perspective and present-mindedness as trained through meditation practice [11]: the former is associated with a strong urge to act in the present moment and is stimulus-oriented, whereas the latter is associated with an observational state associated with more self-control [12]. It has recently been shown that meditation-naïve and meditation-experienced persons after a short mindfulness meditation relatively overestimate duration in the milliseconds-to-seconds range [13,14]. Moreover, students scoring higher on self-reported mindfulness were more accurate in their timing abilities in the milliseconds and multiple-seconds range [12] In a cross-sectional study with experienced mindfulness meditators and matched controls no differences in the timing of short duration (time estimation) was found but meditators experienced less time pressure, more time dilation, and a general slower passage of time (time awareness) [15].

The reported connections between the time dimensions, especially between the present-perspective and time estimation/time awareness, however, are rather unsystematic. For example, there are only few studies showing a direct relationship between a psychometrically validated measure of the time perspective such as the Zimbardo Time Perspective Inventory (ZTPI) [16] and measures of time estimation or time awareness. The ZTPI assesses individual characteristics on five dimensions of the time perspective, namely the past-negative, past-positive, present-hedonistic, present-fatalistic, and future. One cross-sectional study directly showed that a greater future perspective in the ZTPI correlated with longer duration reproductions in the seconds’ range [17]. In another study, time estimation in the seconds’ range was weakly correlated with the time perspective, where the younger subject group (ages between 15 and 25) was more accurate in time estimation and had a more pronounced present-perspective; the older group (ages between 35 and 55) had a more pronounced future perspective [18]. In one further study the everyday experiences of more or less punctuality in life did not strikingly relate to judgments of short duration [19]. Regarding the scarcity of research or a lack of reported direct relationships between the three aspects of time, we wanted to compare the individual time perspective with the subjective awareness of the passage of time. A second aim was to assess the awareness of passing time across age groups. The feeling that time speeds up as we grow older is an impression most people report [20,21,22]. However, only in recent years it has been shown that—at least for populations in industrialized nations—the subjective speed of the passage of time, especially the passage of the last 10 years, indeed speeds up with increasing age [23,24,25]. Interestingly, it is the retrospectively judged time interval of the last 10 years and not the last year, the last month or week that is sensitive for age differences [26].

Retrospective judgements of time are thought to rely on memory processes, *i.e.*, the more contextual changes have been stored in memory the longer duration seems in retrospect [27,28]. An increasing subjective speed of the passage of time when we get older could be explained through the amount of contextual changes stored in episodic memory over that time span; that is, when we get older, due to the fact that we become more experienced and have more routines in life, the novelty of life events diminishes and these events are stored in memory with less emotional strength [22]. This intuitive notion however still has to be proven empirically and there is some debate about the validity and the underlying factors contributing to this widely held notion [20,26,29].

With our study we aim to assess the typically experienced passage of time over longer intervals; thus we are investigating retrospective judgements of subjective time (time awareness). Perception of duration in the seconds-to-minutes range (time estimation) is thought to be governed by specific time keeping systems activated when one is prospectively judging duration, *i.e.*, during a short time interval which one is experiencing at present [27]. In studies on prospective time perception in the multiple-second range results point to age-related differences that are attributable to decreased attention regulation capacity with increasing age [30].

To these two ends, (1) the potential relationship between the time perspective and time awareness; and (2) the question of factors influencing the subjective passage of time over the life span, we conducted an online survey. Regarding the time perspective we employed the ZTPI [16] and additionally used the factor “presence” of the Freiburg Mindfulness Inventory (FMI) [31] as measure of the “holistic presence” time perspective [32]; for the recent development of a positive presence scale, the present-eudaimonic scale, see [33]). That is, the ZTPI contains the hedonistic and fatalistic present perspective, but a different form of presence related to the mindful awareness of the present moment is an additional form of present perspective, especially interesting for the study of subjective time [11,34,35]. In addition, we employed a “balanced time perspective” as calculated from the five dimensions of the ZTPI [36,37]. Regarding typical time awareness in everyday life, questions regarding “time pressure”, “time expansion/boredom”, “routines in life”, and “how the passage of time typically passes” were posed [23]. Moreover, to address the question of age-related differences concerning the subjective passage of time over past time intervals of the last week, month, year, and ten years, additional questionnaires were employed, namely the scales for experiencing emotions (SEE) [38,39], a short form of the five factor personality inventory NEO-FFI (BFI-K; Big Five Inventory—Kurzform [40]) of the five factor personality inventory (the NEO-FFI), and the Barratt Impulsiveness Scale (BIS-11) [41]. Strong emotions in healthy individuals such as with trait-anxiety lead to a slower passage of felt time [42]; impulsive individuals more often overestimate duration and complain that time passes too slowly [5]. Relating to the two open questions mentioned above we aimed at conducting this exploratory study: (1) associating for the first time inventories relating to the time perspective with measures of time awareness; and (2) investigating potential factors related to age-related differences in retrospective judgments of various life spans.

## 2. Method

### 2.1. Participants

A total of 423 individuals between the ages of 17 and 81 participated in the study. Data from 389 subjects were collected through LimeSurvey (Version 1.92). These subjects were contacted through email distribution lists of the authors and of the authors’ institutions as well as social media (Facebook, Twitter). A paper version of the survey was filled out by 34 individuals (8% of the total) who were recruited in person. Mean age of subjects was 34.2 years (S.D.: 15.6 years); 302 (71.4%) were women and 121 (28.6%) were men. The different age groups were represented as follows: <30 years: *n* = 248; 30 to 39 years: *n* = 51; 40 to 49 years: *n* = 47; 50 to 59 years: *n* = 31; 60 to 69 years: *n* = 29; ≥70 years: *n* = 17. The number of paper versions distributed per age group was: <30 years: *n* = 1; 30 to 39 years: *n* = 2; 40 to 49 years: *n* = 9; 50 to 59 years: *n* = 5; 60 to 69 years: *n* = 8; ≥70 years: *n* = 9. Regarding educational background, three participants had not yet achieved a school qualification; 12 participants had the basic required level of education (nine years of primary school; “Hauptschule”); 21 had an intermediate level of schooling (four years of primary school, six years of secondary modern school; “Realschule”), 159 had finished grammar school (four years of primary school, nine years of grammar school, “Gymnasium”); 228 had a university or college degree. The investigation was conducted according to the principles expressed in the Declaration of Helsinki and in accordance with relevant institutional and national guidelines and regulations, that is, the guidelines of the German Psychological Society and the regulations of the Chemnitz University of Technology.

### 2.2. Materials

#### 2.2.1. Zimbardo Time Perspective Inventory (ZTPI)

The German version [43] of the ZTPI [16] contains 56 five-point items ranging from 1 (very untrue) to 5 (very true). Divided into five subscales, an individual reports on his or her emphasize of the time dimensions: past-negative (“I often think about the bad things that have happened to me in the past”), present-hedonistic (“I take risks to put excitement in my life”), future (“I am able to resist temptations when I know that there is work to be done”), past-positive (“Happy memories of good times spring readily to mind”), and present-fatalistic (“Because things always change, one cannot foresee the future”). From the five dimensions of the ZTPI an ideal profile of a balanced time perspective (BTP) can be derived which contains high scores on the past positive time perspective, moderately high scores on the future and the present hedonistic time perspective, and low scores on the past negative and the present fatalistic time perspective (see Zimbardo and Boyd’s website http://www.thetimeparadox.com/surveys/) [36]. Following the reasoning of Stolarski, Wiberg, and Osin [36] for each individual an indicator regarding BTP can be calculated which represents the deviation from the balanced time perspective (DBTP) as measure of how far an individual’s empirical values across the five dimensions are from this ideal value. Suggested optimal values for the BTP are taken as percentiles from the ZTPI dimensions (from Zimbardo and Boyd’s website address). The root of the squared deviations of individual scores from the ideal profile points across the five dimensions is calculated (for the exact equation, see [36]). The lower the DBTP, the closer an individual is to the ideal profile of a balanced time perspective.

#### 2.2.2. Freiburg Mindfulness Inventory (FMI)

The FMI [31,44] contains 14 four-point items with answer categories ranging from 1 (rarely) to 4 (almost always) which evaluate mindfulness on the basis of a two-factor structure. The two factors are “presence” as ability to attend to the present moment (“I am open to the experience of the present moment”) and “acceptance” as non-judgmental attitude (“I am patient with myself when things go wrong”). The factor presence is of special interest as it is conceptually discussed as representing the “holistic presence” as a form of attending mindfully to the “here and now” [32].

#### 2.2.3. Scales for Experiencing Emotions (SEE)

The SEE [38,39] assesses the awareness, appraisal and regulation of one’s emotions with 42 five-point items with answer categories ranging from 1 (very untrue) to 5 (very true). The items are assigned to seven subscales covering: (1) accepting one’s own emotions; (2) experiencing overwhelming emotions; (3) experiencing lack of emotions; (4) bodily symbolisation of emotions; (5) imaginative symbolisation of emotions; (6) experiencing regulation of emotions; (7) experiencing self-control. Since episodic memory processing of encoding and retrieval is closely linked with emotional processes [45], we assessed whether subscales of experiencing emotions could be related to the retrospective judgement of time spans which are supposed to be mediated by memory (see Introduction).

#### 2.2.4. The Barratt Impulsiveness Scale (BIS-11)

The German version [46] of the BIS-11 [41] consists of 30 four-point items with answer categories ranging from 1 (rarely) to 4 (almost always). The resulting subscales are non-planning impulsivity (“I plan tasks carefully”), motor impulsivity (“I do things without thinking”), and attention/cognition impulsivity (“I concentrate easily”).

#### 2.2.5. Big Five Inventory—Short Form (BFI-K)

A short form of 21 five-point items with answer categories ranging from 1 (very untrue) to 5 (very true) for the assessment of the five factors of personality, namely neuroticism, extraversion, openness, agreeableness, and conscientiousness, was used (BFI-K [40]).

#### 2.2.6. Subjective Time Questionnaire

The subjective time questionnaire [23] consists of questions concerning (1) retrospective judgments of the passage of past time intervals and (2) the subjective passage of time typically experienced. (1) Retrospective judgment of past time intervals is assessed with four questions asking how fast *last week*, *last month*, *last year*, and the *past 10 years* have passed. We used visual analogue scales with a slider covering 100 units between the two end points: “very slowly” and “very fast”. (2) Everyday time experience is assessed with 15 statements concerning subjective judgements of time with assigned values of 0 (strong rejection) to 4 (strong approval). The judgments refer to the feeling of *time pressure* (eight statements, e.g., “I often think that time is running out”), to the feeling of *time expansion/boredom* (five statements, e.g., “My time is not filled”) and the feeling of *routines in life* (two statements, e.g., “My everyday life is filled with routine”). We calculated a mean value for the statements on “time pressure”, “time expansion”, and “routines”. Finally, we asked the question of *how the passage of time typically passes* using the slider covering 100 units on a visual analogue scale with the two end points “very slowly” and “very fast”.

### 2.3. Statistics

For the first part of the study, to assess the relationship between the time perspective and the subjective passage of time (time awareness), we first inter-correlated all variables concerning the five dimensions of the ZTPI, the presence subscale of the FMI, and age. Moreover, a series of correlations were conducted to assess the relationship between the balanced time perspective and measures of time awareness as well as between the balanced time perspective and the trait variables. The latter relationship has recently been subjected to research [36,37] and this will be addressed at the end of the results section. Then we conducted a series of step-wise regression analyses. In block 1 of the regression models we had age, sex, and type of data acquisition (online survey = 1; paper version = 2) as independent variables. That is, in addition to age and sex we control for variation attributable to type of data acquisition. After controlling for the variance explained by the data acquisition method, effects of independent content-related variables are interpretable independent of type of method used. The five time dimensions of the ZTPI and the “presence” subscale of the FMI were selected as independent variables in block 2 of the regression model. The dependent variables concerned the typical perception of the passage of time, retrospective judgment of past time intervals, the feeling of time pressure, time expansion/boredom, and routines in life.

For the second part of the study, to assess influences on the phenomenon of an increasing speed of time with advancing age, we conducted step-wise regression analyses between the variables of interest as independent variables (emotion, impulsiveness, and personality) and the retrospective judgements of the last week, the last month, the last year, and the last ten years as dependent variables. Again, in block 1 of the regression model age, sex, and data acquisition method were selected as independent variables, and block 2 of the regression model consisted of the content-related independent variables.

For each correlation and regression analysis significance levels were set to *p* < 0.05. The false discovery rate (FDR) method, a multiple comparisons correction procedure by Benjamini and Hochberg [47], was used to control for multiple tests in the correlation analysis. In the regression analyses we adjusted for the number of regressions analyses conducted. Only relationships that are significant after alpha correction will be discussed, significance levels of 5%, 1%, and 0.1% are nevertheless all reported in the tables. Cronbach’s alpha for the used subscales from the study sample and from German validation studies as well as factor analysis confirmation statistics are presented in Table A1 in the Appendix.

## 3. Results

### 3.1. Inter-Correlations between Dimensions of the Time Perspective

Age is negatively correlated with both the past positive (*r* = −0.158, *p* < 0.001) and the past negative perspective (*r* = −0.214, *p* < 0.0001) of the ZTPI (see Table 1), that is, the older participants were, the less pronounced the two past perspectives were represented. Factor age correlated positively with the presence subscale of the FMI (*r* = 0.118, *p* < 0.015): elder people report to have a stronger present perspective. Present perspective—conceptually thought of as “holistic presence”—positively relates to the past positive perspective (*r* = 0.167, *p* < 0.001) and the present hedonistic perspective (*r* = 0.342, *p* < 0.0001), and negatively with the past negative perspective (*r* = −0.211, *p* < 0.0001). The inter-correlations of the ZTPI subscales confirm the validity study of the ZTPI (Zimbardo and Boyd [16], p. 1276): for example, the future perspective is negatively correlated with the present hedonistic (*r* = −0.360, *p* < 0.0001) and the present fatalistic perspective (*r* = −0.290, *p* < 0.0001); the present hedonistic and the present fatalistic perspective are positively related (*r* = 0.333, *p* < 0.0001), the past positive and the past negative perspectives are negatively correlated (*r* = −0.263, *p* < 0.0001). Mean values of all subsales across subjects and mean values per age group are listed in Table A4 in the Appendix.

**Table 1:** Inter-correlations between the five time dimensions of the ZTPI, the presence subscale of the FMI, and age.

Time Perspective	Future	Past Positive	Past Negative	Present Hedonistic	Present Fatalistic	Presence FMI
Future	1					
Past positive	0.048	1				
Past negative	−0.019	−0.263 ***^,**a**^	1			
Present hedonistic	−0.360 ***^,**a**^	0.187 ***^,**a**^	−0.026	1		
Present fatalistic	−0.290 ***^,**a**^	−0.035	0.372 ***^,**a**^	0.333 ***^,**a**^	1	
Presence FMI	0.021	0.167 ***^,**a**^	−0.211 ***^,**a**^	0.342 ***^,a^	0.025	1
Age	0.090	−0.158 ***^,**a**^	−0.214 ***^,**a**^	−0.026	−0.026	0.118 *^,**a**^

Significant correlation coefficients: * *p* < 0.05; *** *p* < 0.001; **^a^** significant after FDR-adjustment.

### 3.2. Time Perspective as Predictor of Time Awareness and Retrospective Life Spans

Only one time perspective is significantly related to the subjective passage of time of past intervals: the more present hedonistic participants reported to be, the faster the last week passed by (β = 0.172, *p* < 0.003) (Table 2). As expected, age predicted the time experience of the last ten years (β = 0.292, *p* < 0.0001); the older participants were, the faster the last ten years passed subjectively. This finding will be further analysed below. Several significant regression coefficients indicate that the time perspective as trait predicts experiences of time pressure, time expansion/boredom, routine, and the typically experienced passage of time (Table 3). A pronounced past negative perspective predicts the feeling of time pressure (β = 0.260, *p* < 0.0001), as well as the opposite, time expansion/boredom (β = 0.272, *p* < 0.0001), and the feeling of more routine in life (β = 0.141, *p* < 0.011). The present hedonistic perspective is related to the sense of having less routines in life (β = −0.281, *p* < 0.0001). The future perspective predicts a faster experienced everyday passage of time (β = 0.141, *p* < 0.011). Moreover, age predicts the feeling of less time pressure (β = −0.167, *p* < 0.002).

**Table 2:** Standard regression coefficients (β) with the five time dimensions of the ZTPI (block 2), the presence subscale of the FMI (block 2), and age, sex, and data acquisition method (block 1) as independent variables separately included in step-wise regression models using as dependent variables the feeling of the passage of time concerning the past intervals of last week, last month, last year, and the last 10 years.

Variables Block 1, 2	Last Week	Last Month	Last Year	Last 10 Years
1. Age	−0.084	−0.126 *	−0.105	0.292 ***^,**a**^
1. Sex	−0.019	−0.020	−0.101	−0.084
1. Data acquisition	0.151 **^,**a**^	0.162 **^,**a**^	0.151 **^,**a**^	0.095
2. Future	0.065	0.051	0.054	0.055
2. Past positive	0.040	0.011	−0.002	−0.059
2. Past negative	0.101	0.023	−0.047	0.126 *
2. Present hedonistic	0.172 **^,**a**^	0.105	−0.020	0.031
2. Present fatalistic	−0.001	−0.020	0.023	0.007
2. Presence FMI	−0.017	−0.001	0.014	−0.032
R^2^ model including all variables	0.054 *	0.035	0.038	0.134 *

Significant regression coefficients: * *p* < 0.05; ** *p* < 0.01; *** *p* < 0.001; **^a^** significant after alpha adjustment for four regression analyses (*p* < 0.0125).

**Table 3:** Standard regression coefficients (β) with the time dimensions of the ZTPI (block 2), the presence subscale of the FMI, age, sex, and data acquisition method (block 1) as independent variables separately included in step-wise regression models using as dependent variables the feeling of time pressure, time expansion, routine, and the typical passage of time.

Variables Block 1, 2	Time Pressure	Time Expansion/Boredom	Routine	Typical Passage of Time
1. Age	−0.167 **^,**a**^	−0.109 *	−0.013	0.005
1. Sex	0.007	0.028	−0.033	0.010
1. Data acquisition	0.083	−0.017	0.065	0.096
2. Future	−0.038	−0.095	0.078	0.141 **^,**a**^
2. Past positive	0.000	−0.023	0.003	−0.002
2. Past negative	0.260 ***^,**a**^	0.272 ***^,**a**^	0.141 *^,**a**^	−0.012
2. Present hedonistic	0.072	0.012	−0.281 ***^,**a**^	0.118 *
2. Present fatalistic	0.082	0.059	0.083	0.038
2. Presence FMI	−0.122 *^,**a**^	−0.044	0.029	−0.012
R^2^ model including all variables	0.170 ***^,**a**^	0.146 ***^,**a**^	0.1 ***^,**a**^	0.035

Significant regression coefficients: * *p* < 0.05; ** *p* < 0.01; *** *p* < 0.001; **^a^** significant after alpha adjustment for four regression analyses (*p* < 0.0125).

Regarding the notion of a balanced time perspective (BTP), the deviation from the BTP (DBTP) as independent variable (in block 2 of the step-wise regression model; age, sex and type of data acquisition controlled for in block 1) predicts the passage of the last 10 years (β = 0.125, *p* < 0.007) (Table 4): the more balanced the time perspective, the longer the last 10 years seem to have lasted. Moreover, the DBTP predicts the feeling of time pressure: the less balanced the time perspective, the more time pressure (β = 0.267, *p* < 0.0001). The DBTP predicts time expansion/boredom: the less balanced the time perspective, the more time pressure (β = 0.280, *p* < 0.0001). The DBTP predicts routines in life: the less balanced the time perspective, the more routines are experienced (β = 0.151, *p* < 0.002) (Table 5).

**Table 4:** Standard regression coefficients (β) with the deviation of the balanced time perspective (DBTP) (block 2), age, sex, and data acquisition method (block 1) as independent variables) separately included in step-wise regression models using as dependent variables the feeling of the passage of time concerning the past intervals of last week, last month, last year, and the last 10 years.

Variables Block 1, 2	Last Week	Last Month	Last Year	Last 10 Years
1. Age	−0.081	−0.131 *	−0.106	0.272 ***^,**a**^
1. Sex	−0.021	−0.020	−0.100 *	−0.084
1. Data acquisition	0.149 **^,**a**^	0.161 **^,**a**^	−0.152 **^,**a**^	0.092
2. DBTP	0.032	0.006	−0.013	0.125 **^,**a**^
R^2^ model including all variables	0.020	0.025	0.033	0.122 **^,**a**^

Significant regression coefficients: * *p* < 0.05; ** *p* < 0.01; *** *p* < 0.001; **^a^** significant after alpha adjustment for four regression analyses (*p* < 0.0125).

**Table 5:** Standard regression coefficients (β) with the deviation of the balanced time perspective (DBTP) (block 2), age, sex, and data acquisition method (block 1) as independent variables separately included in step-wise regression models using as dependent variables the feeling of time pressure, time expansion, routine, and the typical passage of time.

Variables Block 1, 2	Time Pressure	Time Expansion/Boredom	Routine	Typical Passage of Time
1. Age	−0.236 ***^,**a**^	−0.186 **^,**a**^	−0.024	0.008
1. Sex	0.016	0.040	−0.049	0.002
1. Data acquisition	0.088	−0.025	0.076	0.114 *
2. DBTP	0.267 ***^,**a**^	0.280 ***^,**a**^	0.151 **^,**a**^	−0.008
R^2^ model including all variables	0.122 ***^,**a**^	0.115 ***^,**a**^	0.030 **^,**a**^	0.014

Significant regression coefficients: * *p* < 0.05; ** *p* < 0.01; *** *p* < 0.001; **^a^** significant after alpha adjustment for four regression analyses (*p* < 0.0125).

### 3.3. Predictors of the Subjective Passage of the Past 10 Years

Next we assessed whether we could find predictors for the only elapsed time span which is sensitive for age differences—the last 10 years. That is, age is a predictor for the subjective passage of time over the last ten years (Table 2; β = 0.292, *p* < 0.0001). The emotion subscales of the SEE “emotion regulation” (β = −0.159, *p* < 0.004) is predictive of the subjective impression of passage of time over the last ten years (Table 6): the more emotion regulation the slower the last ten years passed. None of the time dimensions of the ZTPI or the presence subscale of the FMI is related to the feeling of passing time over the last 10 years (Table 2). The subjective time judgments of feeling time pressure (β = 0.117, *p* < 0.018), and the typical subjective passage of time (β = 0.160, *p* < 0.002) are predictive of an increased subjective speed of time over the last ten years (Table 7). None of the five subscales related to the short form of the NEO-FFI, the BFI-K, was related to the passage of the last 10 years (Appendix Table A2). None of the impulsiveness subscales of the BIS was related to the retrospective 10-year scale of time awareness (Appendix Table A3).

### 3.4. Relations between a Balanced Time Perspective and Trait Variables

A balanced time perspective in general is correlated with mindfulness (FFA sum score; *r* = −0.361, *p* < 0.0001): the more mindful, the smaller the deviation from the ideal time perspective profile; the DBTP is related to impulsiveness (BIS sum score; *r* = 0.193, *p* < 0.0001): the more impulsive individuals, the larger the deviation from the ideal time perspective. The DBTP correlates significantly with several scales for experiencing emotions (SEE): (1) the more acceptance of one’s own emotions, the lower the deviation from a balanced time perspective (*r* = −0.320, *p* < 0.0001); (2) the more one experiences overwhelming emotions, the larger the DBTP (*r* = 0.534, *p* < 0.0001); (3) experiencing a lack of emotions is correlated with higher DBTP values (*r* = 0.298, *p* < 0.0001); (4) the bodily symbolisation of emotions also relates positively to DBTP (*r* = 0.145, *p* < 0.003); (5) the imaginative symbolisation of emotions is not significantly correlated with the balanced time perspective (*r* = 0.104, *p* < 0.032; n.s. after alpha adjustment); (6) experiencing emotion regulation (*r* = −0.248, *p* < 0.0001) and (7) experiencing self-control (*r* = 0.141, *p* < 0.004) are both related to less deviation from the ideal time perspective. Finally, four of the five factors of personality (BFI-K) relate to the balanced time perspective score DBTP. Higher values in neuroticism are related to higher values in DBTP (*r* = 0.495, *p* < 0.0001); extraversion (*r* = −0.286, *p* < 0.0001), friendliness (*r* = −0.178, *p* < 0.0001), and conscientiousness (*r* = −0.249, *p* < 0.0001) are negatively correlated with deviations from the balanced time perspective. That is, a more balanced time perspective is more likely to be found in individuals who are less extroverted, friendlier, and more conscientious. Age is not correlated with the DBTP score (*r* = −0.046, *p* < 0.345).

**Table 6:** Standard regression coefficients (β) in a step-wise regression model with the seven subscales of the emotion scale SEE (block 2), age, sex, and data acquisition method (block 1) as independent variables and the subjective passage of time concerning the last 10 years as dependent variable.

Variables of the SEE Block 1, 2	Subjective Passage of Time Past 10 Years
1. Age	0.259 ***
1. Sex	−0.051
1. Data acquisition	0.088
2. Accepting one’s own emotions	0.103
2. Experiencing overwhelming emotions	0.034
2. Experiencing lack of emotions	0.096
2. Bodily symbolisation of emotions	−0.039
2. Imaginative symbolisation of emotions	−0.013
2. Experiencing regulation of emotions	−0.159 **
2. Experiencing self-control	−0.039
R^2^ model including all variables	0.146 **

Significant regression coefficients: ** *p* < 0.01; *** *p* < 0.001.

**Table 7:** Standard regression coefficients (β) in a step-wise regression model with time awareness measures (block 2), age, sex, and data acquisition method (block 1) as independent variables); the subjective passage of time of the last 10 years is the dependent variable.

Variables of Present Time Experience Block 1, 2	Subjective Passage of Time Past 10 Years
1. Age	0.303 ***
1. Sex	−0.077
1. Data acquisition	0.060
2. Time pressure	0.117 *
2. Time expansion/boredom	−0.004
2. Routine	0.089
2. Typical passage of time	0.160 ***
R^2^ model including all variables	0.147 ***

Significant regression coefficients: * *p* < 0.05; *** *p* < 0.001.

## 4. Discussion

The two study parts assessing: (1) influences of the individual time perspective on time awareness and (2) psychological factors influencing the perception of the passage of time over the last ten years revealed the following results:

The mindful-oriented present perspective of the FMI correlated moderately and positively with the ZTPI subscales of hedonistic presence and the past positive perspective; there is a negative association between the mindful-present perspective and the past negative perspective. The holistic presence thereafter is associated with enjoying being and acting in the here and now as well as looking back into the past with positive feelings. Also with increasing age participants have a stronger mindful present-perspective. With one exception, the dimensions of the time perspective did not relate to the retrospective judgments of past intervals; individuals who are more present-hedonistic felt a faster passage of the last week. Age, as repeatedly found, was a predictor of the perception of the passage of time over the last ten years: the older participants the faster time passed by. Several effects between the dimensions of the time perspective and everyday experience of time were detected: a faster speed of the passage of time was predicted by a more pronounced future perspective; a pronounced past negative perspective is related to more time pressure, time expansion/boredom, and the feeling of more routine in life; past negative feelings thus are overall related to negative impressions of the passage of time. Fitting into the overall picture, being a more present-perspective hedonist is related to having less routine in life. With increasing age participants experienced less time pressure and less time expansion/boredom, *i.e.*, they have generally more positive feelings about time. However, when inspecting age-group differences descriptively (Table A4), less time expansion/boredom is mostly experienced between the ages of 30 and 59 years, the life span when individuals are most committed to work; decreased time pressure is most pronounced when people are 60 years and older, *i.e.*, when they are more likely retired (for similar results, see [23]).

What are factors influencing the retrospective passage of time over the last 10 years, the time interval of our lives that speeds up as we get older? It is the ability to regulate emotions and a more balanced time perspective which is associated with a slower passage of this time span. Relating to everyday time experiences, feeling time pressure and a general faster passage of time are both related to the retrospective faster passage of the last ten years.

The experience of time can be inter-individually characterized. The personal pattern of how the time perspective is represented influences—as we show here—everyday experience of the subjective passage of time. Individuals with a stronger future perspective have the feeling that time passes typically more quickly. The relation between the future perspective and a faster subjective passage of time is understandable in the context of models of time perception. Subjective time emerges through the perception of the self across time as an enduring and embodied entity [48,49]: as has empirically been shown, intertwined affective and interoceptive states of the body create our experience of duration [50,51]. Moreover, an increased awareness of oneself and the surrounding world coincides with an increased awareness of time over longer intervals in retrospect—leading to the feeling that time passes more slowly [52]. Although in our study we did not find a relationship between the holistic present perspective and the typical passage of time (though a more mindful present-perspective is related to less time pressure), a more pronounced future perspective at the expense of the present perspective thereafter would lead to a general faster passage of subjective time.

The past negative perspective is overall related to a negative sense of time, that is, to the opposed feelings of more time pressure and to more boredom. Following Zimbardo and Boyd [32] (p. 88), in their description of this perspective, the attitudes about past events, which matter more than the events themselves, are “key to the development of gratitude, which allows you to appreciate your life in the present.” Positive attitudes towards the past are also related to greater satisfaction with present life. Emotional distress, in contrast, is related to alterations in subjective time, namely to both extreme situations where time passes too slowly or when there is not enough time [42,53,54]. Conforming to earlier studies on successful aging [55], people with increasing age do not become more past oriented. To the contrary, as we show in our sample with individuals with an upper age range of 81, age is actually negatively related to both the negative and the positive past perspective; in accordance with this positive appraisal of aging effects, older subjects have a stronger present perspective.

Our results confirm previous studies showing that the retrospectively judged time interval of the past 10 years and not the last year, the last month or week is associated with age. With increasing age individuals feel that time is passing more quickly [23,24,25]. Memory-related explanations pertaining to routine and to the loss of novelty of events as we become older have theoretically been postulated [22]. The fact that experiencing more routine in life is linked to a faster passage of the last decade in our study additionally validates this claim; routine has been shown to speed up subjective time [28]. Here we show how emotions—strongly linked to memory encoding and retrieval [45] as well as to memory for duration [56]—are predictive of the feeling of the passage of the last ten years. Merely being exposed to more emotions, such as registered by many subscales of the inventory for experiencing emotions (SEE), does not lead to a slower passage of time of the past ten years.It is the subscale of emotional self-regulation that leads to a slower passage of subjective time over this time interval. What these results point to, regarding the memory-load model of retrospective time, is that the mere passive feelings of emotions themselves are not sufficient to encode and retrieve more efficiently memory contents—and thus expand subjective time. It is the competence to voluntarily and actively regulate mood states and emotional reactions. As shown in another validation study, the capacity of emotion regulation is negatively correlated with neuroticism, stress, depression, and trait-anxiety and positively correlated with life satisfaction; moreover, it is positively linked to general problem solving, self-esteem, and the amount of social contacts, among other variables [39]. That is, emotion regulation is a proxy for having variable coping strategies, emotional and instrumental, as well as for the sense of self-efficacy. This form of emotional and social intelligence thus is a means to feel a subjective slower passage over the last ten years. Individuals who feel a faster passage of the past decade also feel more time pressure and a generally faster speed of the passage of time. According to our results, emotionally competent individuals have the propensity to expand their subjective life time.

Another factor that leads to a slower passage of the last 10 years is having a balanced time perspective, meaning that an individual loads high on the past positive time perspective, has moderately high scores on the future and the present hedonistic time perspective, and shows low scores on the past negative and the present fatalistic time perspective [32,36]. Moreover, a balanced time perspective, which is age-independent, is correlated with more mindfulness, less impulsivity, a better handling of emotions, and with more positive personality traits (friendliness and less neuroticism). A person who is more balanced in his or her time perspective is future orientated but can also savor the moment and can positively learn from the past. Our empirical findings strongly speak in favor of the idea that a balanced time perspective is an indication of individual happiness and health [32]—let alone the existential fact that for individuals with a balanced time perspective life time passes more slowly.

Limitations of this study are the education and age bias in the study sample. That is, a large proportion of subjects were comparably highly educated and young, a bias which is typically seen in academic studies capitalizing on easy access to the student population. However, our results on retrospective judgments of duration are very similar to outcomes of former studies having a more balanced age and education sample. Therefore, we think that the found relations between the time perspective and time awareness as well as the age differences are representative for people in industrialized nations. Finally, one methodological issue has to be mentioned: retrospective judgments of duration are supposed to rely on memory retrieval processes pertaining to episodic memories of time spans lived [23,24]. However, this relationship has still not been demonstrated directly through measures of episodic memory and by comparing memory retrieval for various life spans between different age groups. This approach would amount to a huge logistical endeavor but would probe directly whether our assumptions are valid, namely that subjective life expands with a greater amount of memories present for a given interval of our life.

Another open question pertains to the fact that the last 10 years, and not the last year, is sensitive to age differences, and that emotion regulation and a balanced time perspective are related to the felt passage of the last 10 years, but not to the last year. Adults indeed often complain how the last year has again passed by so quickly. One possible answer would be that in our study individuals compared the last interval (1 year/10 years) with the preceding same interval, and the felt contrast between the last 10 years and the preceding 10 years is larger than the contrast between the last year and the preceding year. However, these methodological issues have to be addressed in future studies.

## 5. Conclusions

We found several meaningful connections between the time perspective as trait and the awareness of time. That is, the mindful-present dimension relates to less time pressure, the present-hedonistic dimension to less routine and to a faster passage of the last week; the past negative dimension predicts negative experiences of time, *i.e.*, more time pressure, boredom, and routine; the more future oriented an individual, the faster the typical passage of time is experienced. Moreover, the ability to regulate emotions—a proxy for emotional intelligence—leads to a relatively more slowly experienced 10 years of one’s life. A similar influence on the retrospective judgment of the previous 10 years was detected with the balanced time perspective, which represents a form of “temporal freedom”. Temporal freedom here would mean to not be stuck in negative ruminations over the past or to be only living hedonistically in the present at the expense of the future. It would also mean not to be too future-oriented at the expense of enjoyable experiences in the present moment. People with a more balanced time perspective experience the past 10 years to have passed more slowly.

## Appendix

**Table A1:** Cronbach’s alpha for each subscale (or entire scale *****) from the study sample and from German validation studies; factor analysis confirmation with eigenvalues above 1.

Questionnaire	Subscale (*n* Items)	Cronbach’s Alpha Study Sample/Validation Study	Factor Structure (Confirmatory) Eigenvalues Above 1
BFI-K (21 Items)	extraversion (4)	0.821/0.81	5-factor structure confirmed; explained variance: 58.0%
	agreeableness (4)	0.618/0.67	
	conscientiousness (4)	0.722/0.62	
	neuroticism (4)	0.781/0.65	
	openness (5)	0.703/0.70	
ZTPI (56 Items)	past positive (9)	0.746/-	5-factor structure confirmed; explained variance: 38.3%
	past—negative (11)	0.844/-	
	present—hedonistic (15)	0.802/-	
	present—fatalistic (9)	0.694/-	
	future (13)	0.757/-	
BIS (30 Items)	motor (11)	0.707/0.74 *****	3-factor structure confirmed; explained variance: 34.4%
	planning (11)	0.677/0.74 *****	
	attention/cognition (8)	0.581/0.74 *****	
FFA (14 Items)	presence (5)	0.622/0.69	2-factor structure confirmed; explained variance: 40.6%
	acceptance (9)	0.793/0.77	
SEE (42 Items)	imaginative symbolisation (6)	0.837/0.82	7-factor structure confirmed; explained variance: 56.1%
	emotion regulation (4)	0.676/0.70	
	overwhelming emotions (7)	0.870/0.86	
	bodily symbolisation (8)	0.799/0.80	
	self-control (6)	0.767/0.76	
	accepting one’s emotions (6)	0.858/0.82	
	lack of emotions (5)	0.775/0.70	
Subjective time questionnaire (15 Items)	time pressure (8)	0.813/-	3-factor structure confirmed; explained variance: 56.7%
	expansion/boredom (5)	0.822/-	
	routines in life (2)	0.625/-	

***** In German validation study of the BIS [46] Crobach’s alpha was only calculated for the sum scale.

**Table A2:** Standard regression coefficients (β) in a step-wise regression model with the five subscales of the personality inventory BFI-K (block 2), age, sex, and data acquisition method (block 1) as independent variables, and the subjective passage of the last 10 years as dependent variable.

Variables of the BFI-K Block 1, 2	Subjective Passage of Time Past 10 Years
1. Age	**0.272 *****
1. Sex	−0.068
1. Data acquisition	0.091
2. Extraversion	0.020
2. Openness	−0.007
2. Agreeableness	−0.054
2. Conscientiousness	0.058
2. Neuroticism	0.072
R^2^ model including all variables	0.118

Significant regression coefficients: ******* *p* < 0.001.

**Table A3:** Standard regression coefficients (β) in a step-wise regression model with the three subscales of the impulsiveness scale BIS (block 2), age, sex, and data acquisition method (block 1) as independent variables and the subjective passage of the last 10 years as dependent variable.

Variables of the BIS Block 1, 2	Subjective Passage of Time Past 10 Years
1. Age	**0.279 *****
1. Sex	−0.076
1. Data acquisition	0.097
2. Attention/cognition impulsivity	0.021
2. Motor impulsivity	−0.044
2. Non-planning impulsivity	0.046
R^2^ model including all variables	0.109

Significant regression coefficients: ******* *p* < 0.001.

**Table A4:** Mean values of subsales (S.D.) across subjects and mean values (S.D.) per age group.

Questionnaire Scale	All Subjects	<30 Years	30–39 Years	40–49 Years	50–59 Years	60–69 Years	>70 Years
BFI-K extraversion	3.33 (0.9)	3.30 (0.9)	3.42 (0.9)	3.45 (0.8)	3.37 (0.7)	3.35 (0.7)	3.06 (0.6)
BFI-K agreeableness	3.05 (0.8)	2.99 (0.8)	2.96 (0.8)	3.21 (0.8)	3.15 (0.7)	3.27 (0.6)	3.25 (0.6)
BFI-K conscientiousness	3.65 (0.7)	3.48 (0.9)	3.72 (0.7)	3.83 (0.6)	4.02 (0.6)	4.98 (0.5)	4.03 (0.6)
BFI-K neuroticism	3.15 (0.9)	3.31 (0.9)	2.97 (0.8)	2.82 (0.8)	2.98 (0.8)	3.00 (1.0)	2.86 (0.9)
BFI-K openness	3.97 (0.7)	3.91 (0.7)	4.16 (0.6)	3.93 (0.7)	4.15 (0.8)	3.97 (0.5)	4.00 (0.6)
ZTPI past positive	3.55 (0.6)	3.61 (0.6)	3.64 (0.6)	3.46 (0.6)	3.32 (0.7)	3.28 (0.4)	3.56 (0.4)
ZTPI past—negative	2.80 (0.8)	2.96 (0.8)	2.77 (0.7)	2.47 (0.8)	2.43 (0.8)	2.54 (0.8)	2.74 (0.8)
ZTPI present—hedonistic	3.17 (0.5)	3.19 (0.5)	3.11 (0.6)	3.17 (0.5)	3.16 (0.6)	3.27 (0.4)	3.00 (0.5)
ZTPI present—fatalistic	2.65 (0.6)	2.68 (0.6)	2.59 (0.6)	2.60 (0.6)	2.61 (0.5)	2.59 (0.5)	2.76 (0.9)
ZTPI future	3.55 (0.5)	3.51 (0.5)	3.53 (0.5)	3.57 (0.5)	3.75 (0.5)	3.56 (0.4)	3.68 (0.4)
Deviation of BTP (DBTP)	2.30 (0.7)	2.34 (0.7)	2.23 (0.7)	2.20 (0.7)	2.29 (0.6)	2.23 (0.6)	2.33 (0.9)
BIS motor	2.00 (0.4)	2.02 (0.4)	2.14 (0.4)	1.96 (0.3)	1.96 (0.4)	1.89 (0.3)	1.69 (0.3)
BIS non-planning	2.19 (0.4)	2.20 (0.4)	2.26 (0.4)	2.19 (0.4)	2.12 (0.4)	2.09 (0.4)	1.96 (0.3)
BIS attention/cognition	2.00 (0.4)	2.11 (0.4)	1.99 (0.4)	1.83 (0.3)	1.87 (0.4)	1.75 (0.3)	1.73 (0.3)
FFA presence	2.77 (0.5)	2.73 (0.4)	2.74 (0.4)	2.87 (0.4)	2.79 (0.6)	2.90 (0.5)	2.87 (0.5)
FFA acceptance	2.63 (0.5)	2.55 (0.5)	2.71 (0.4)	2.76 (0.4)	2.72 (0.6)	2.74 (0.5)	2.78 (0.4)
SEE imaginative symbolisation	2.70 (0.9)	2.78 (0.8)	2.70 (0.7)	2.75 (0.9)	2.56 (1.0)	2.30 (0.7)	2.22 (1.0)
SEE emotion regulation	3.05 (0.7)	3.02 (0.7)	3.18 (0.7)	3.14 (0.7)	3.14 (0.7)	2.98 (0.7)	2.85 (0.8)
SEE overwhelming emotions	2.85 (0.8)	3.03 (0.8)	2.63 (0.7)	2.63 (0.8)	2.57 (0.9)	2.55 (0.9)	2.41 (0.8)
SEE bodily symbolisation	3.20 (0.7)	3.22 (0.6)	3.15 (0.6)	3.33 (0.7)	3.33 (0.8)	2.98 (0.5)	2.98 (0.8)
SEE self-control	3.28 (0.7)	3.23 (0.7)	3.29 (0.6)	3.38 (0.6)	3.35 (0.7)	3.23 (0.8)	3.61 (0.6)
SEE accepting one’s emotions	3.61 (0.7)	3.51 (0.7)	3.75 (0.7)	3.75 (0.7)	3.76 (0.7)	3.72 (0.8)	3.85 (0.7)
SEE lack of emotions	2.24 (0.7)	2.20 (0.7)	2.26 (0.8)	2.14 (0.7)	2.35 (0.9)	2.30 (0.6)	2.76 (0.8)
Time pressure	3.37 (0.7)	3.43 (0.6)	3.58 (0.6)	3.41 (0.8)	3.45 (0.8)	2.69 (0.8)	2.88 (0.7)
Expansion/boredom	2.03 (0.8)	2.19 (0.8)	1.86 (0.7)	1.65 (0.7)	1.67 (0.6)	2.07 (0.8)	1.99 (0.7)
Routines in life	3.04 (0.8)	3.06 (0.8)	3.02 (0.8)	3.07 (0.8)	2.82 (0.8)	3.09 (0.8)	3.12 (0.9)
Typical passage of time	67.2 (14)	66.7 (13)	67.2 (15)	67.8 (18)	73.2 (14)	64.6 (20)	66.9 (17)
Passage of last week	71.4 (20)	71.8 (20)	70.0 (22)	72.4 (20)	74.9 (19)	67.7 (19)	67.9 (16)
Passage of last month	73.2 (18)	73.4 (18)	76.9 (19)	71.5 (18)	77.3 (17)	66.6 (21)	66.8 (21)
Passage of last year	75.4 (18)	75.6 (19)	79.0 (18)	73.2 (14)	78.0 (15)	70.5 (21)	71.5 (13)
Passage of last 10 years	65.8 (21)	60.3 (21)	72.1 (21)	72.1 (17)	76.5 (16)	72.7 (16)	77.7 (12)
